# Pilot Study of SATELLITE Education on Nurses’ Knowledge and Confidence toward Assessing and Caring for Female Victims of Sexual Violence

**DOI:** 10.3390/nursrep14020097

**Published:** 2024-05-22

**Authors:** Ratchneewan Ross, Francine Hebert Sheppard, Monir M. Almotairy, Joelle Hirst, Marjorie Jenkins

**Affiliations:** 1 School of Nursing, The University of Louisville, Louisville, KY 40202, USA; joelle.hirst@louisville.edu; 2 School of Nursing, College of Health and Human Sciences, Western Carolina University, Cullowhee, NC 28723, USA; fsheppard@email.wcu.edu; 3 Department of Nursing Administration and Education, King Saud University College of Nursing, Riyadh P.O. Box 642, Saudi Arabia; malmotairy@ksu.edu.sa; 4 Nursing Administration, Cone Health, Greensboro, NC 27401, USA; marjorie.jenkins@conehealth.com

**Keywords:** sexual violence, screening, care guide, feasibility, acceptability, efficacy, knowledge, confidence, pilot study

## Abstract

Sexual violence (SV) can deeply impact victims’ physical and psychosocial well-being. Yet many healthcare providers, including registered nurses (RNs), hesitate to screen patients due to a lack of confidence and knowledge. The SATELLITE Sexual Violence Assessment and Care Guide was developed to address this gap; however, the guide’s educational effectiveness remained untested. This pilot study aimed to assess the feasibility, acceptability, and efficacy of an education program based on the SATELLITE guide among RNs in clinical settings (n = 8), using a pre- and post-test design. Results indicated that the education was not only feasible and acceptable, but also demonstrated the effects as desired with significant increases in RNs’ knowledge and confidence in SV screening and care. The program’s assessment tool was reliable, and participant recruitment was feasible. Based on these findings, it is recommended that the SATELLITE education program be further tested with a larger RN sample and extended to other healthcare providers. Additionally, exploring SATELLITE’s use in different regions, cultural contexts, and healthcare settings would enhance understanding of the program’s broader applicability and effectiveness.

## 1. Background

Sexual violence (SV) represents a profoundly traumatic life event with far-reaching physical and psychosocial impacts on its victims. Sexual violence is defined by the Rape, Abuse & Incest National Network (RAINN) [1] as “an all-encompassing, non-legal term that refers to crimes such as sexual assault, rape, and sexual abuse”. As described by the Centers for Disease Control and Prevention [2], SV is “sexual activity when consent is not obtained or freely given”. SV stands as a preventable yet pervasive public health concern, affecting millions of women of all ages worldwide [3].

Due to underreporting, varying definitions of sexual violence, and differences in data collection methodologies across countries, SV prevalence estimates fluctuate across regions. Although men and women experience SV, women remain at greatest risk with one in three women affected globally [4]. In the United States, it is estimated that an individual is sexually victimized every 68 s, with one out of every six women being affected [5]. 

SV can lead to a myriad of physical and mental health sequelae, exacerbating victims’ feelings of powerlessness and diminishing self-esteem [6]. Physical health outcomes associated with sexual violence include chronic pain, asthma, difficulty sleeping, and digestive problems [6,7,8]. Sexual and reproductive health outcomes may include pregnancy and sexually transmitted infections as well as chronic conditions such as sexual dysfunction, dysmenorrhea, and menorrhagia [8]. Female victims of SV commonly report at least one symptom of post-traumatic stress disorder [6,9]. Furthermore, experiencing child sexual abuse has been linked to various adverse psychosocial outcomes in adulthood, including substance misuse, decreased life satisfaction, and increased risk of suicidal behaviors [10,11,12].

The economic impact of rape, a form of SV identified as the costliest crime in the United States, is staggering, amounting to an estimated $3.1 trillion over victims’ lifetimes [9]. This figure encompasses medical costs, lost earnings, pain, suffering, and diminished quality of life [9]. Based on economic data from 2015, the average lifetime cost of nonfatal child sexual abuse (CSA) is $282,734 per female victim [13].

Nurses hold a unique position within healthcare clinical settings, regularly encountering numerous women whose health may have been impacted by experiences of SV. Given the profound and lasting effects of SV, it is imperative nurses possess the necessary knowledge and confidence to promptly identify patients with a history of SV victimization, enabling timely referral and intervention to mitigate the adverse consequences of this trauma. Providing such high-quality, equitable nursing care not only enhances patient well-being but also aligns with key recommendations outlined in “The Future of Nursing 2020–2030” [14] as well as the revised nursing scope and standards of practice by the American Nurses Association [15].

The World Health Organization, American College of Obstetricians and Gynecologists (ACOG), and American Medical Association recommend routinely screening all women for a history of sexual violence [16]. Although domestic violence screening education [17,18,19] and numerous SV screening tools are available [20], discussions between healthcare professionals, including registered nurses (RNs), and victims often occur sporadically. Despite state and national screening mandates, a study conducted in California found that only 14% of healthcare professionals, including RNs, consistently screen female patients, while a third of respondents stated they rarely or never screen them [21]. Additionally, the acceptability of screening among healthcare providers in the United Kingdom (UK) did not meet the National Screening Committee criteria for a screening program, as reported by Felder et al. [22]. This finding is consistent with a systematic review and meta-analysis by O’doherty et al. [23], which identified insufficient screening and referrals in healthcare settings. This irregular screening and assessment often stem from RNs’ lack of knowledge and confidence regarding SV assessment, including how to intuitively identify and respond to distress cues, how to broach the subject sensitively, and how to respond effectively when SV experiences are disclosed [24]. In addition to RNs, other healthcare providers also lack this confidence and knowledge. For example, the American Academy of Physician Assistants [25] reports 45.6% of clinically practicing physician assistants surveyed (n = 1633) had never received training on the care of victims of SV, and 51.9% of respondents did not feel adequately prepared to treat or refer SV victims.

To address these barriers, the SATELLITE Sexual Violence Assessment and Care Guide (SATELLITE) was developed to assist healthcare providers in overcoming barriers, including discomfort and reluctance to screen and intervene, when patients disclose SV experiences [26]. The SATELLITE guides healthcare providers “through the process from setting the context for screening, the screening itself, and the interventions after a positive screen finding, including specific questions to be asked and statements to be made by the practitioner in providing care for an SV survivor” [26]. The acronym “SATELLITE” represents Setting, Approach/Ask/Assess, Treat (Thank, Reassure, Empower, Assist, and Take notice), Evaluate, Laws and Legal Implications, and Thorough Education and documentation [26]. Since its inception in 2009, the SATELLITE’s content has been integrated into various textbooks and continuing education programs for healthcare providers, including physician assistants, nursing students, RNs, and advanced practice nurses (APNs) [27,28,29]. However, its effectiveness in enhancing nurses’ knowledge and confidence regarding SV assessment and care in the clinical setting remains unexplored.

Therefore, this pilot study aimed to assess the feasibility, acceptability, and efficacy of the SATELLITE education program in enhancing nurses’ knowledge and confidence in SV assessment and care for SV survivors. This study addresses critical questions: Can the SATELLITE education program be implemented as planned? Are the instruments reliable? Will key stakeholders embrace the education? Does the education produce the desired effects on RNs’ knowledge and confidence in SV assessment and care provision for SV victims? Findings from this study may serve as a foundation for future larger-scale investigations and the utilization of SATELLITE education as a tool to empower nurses in SV assessment and the provision of appropriate care for SV survivors in clinical settings.

## 2. Method

### 2.1. Design

The team employed a single-group, educational intervention utilizing a pre- and post-test design, structured upon the Training Effectiveness model proposed by the Centers for Disease Control and Prevention (CDC) [30]. According to this model, assessing the program’s efficacy is crucial, entailing pre- and post-training evaluations of participants’ knowledge and confidence aligned with the program’s learning objectives [30]. Post-program learner satisfaction was not assessed, as evidence suggests it does not correlate with training effectiveness [30]. For designing the SATELLITE education, the team drew upon the Andragogy learning theory [31], which emphasizes that adult learners are motivated by their own needs and aspirations for career progression and success. This theory suggests that adults learn most effectively through accumulated life experiences [31]. Thus, the delivery of materials in this pilot study heavily incorporated discussions based on the learners’ past experiences with SV screening and patient care. An example prompt question included “Please share with the team your methods for SV screening and patient care experiences related to SV”.

### 2.2. Setting and Sample

Convenience sampling was used. Inclusion criteria included RNs *(n* = 11) who worked at the women’s health center at a large healthcare system in the southeastern region of the United States where the researchers were invited by the unit manager to present SATELLITE education program (as continuing education) as part of the unit monthly meeting. There were no exclusion criteria. Out of 11 RNs, 3 had to leave during the presentation due to patient emergencies and left 8 RNs (6 RNs and 2 advanced practice nurses) who completed both pre- and post-intervention surveys, indicating 100% response rate. All participants had screened patients for SV prior to the SATELLITE education session and had worked as nurses for 14–40 years (median = 20 years).

### 2.3. Instruments 

#### 2.3.1. SATELLITE Education Program

**Figure 1:**
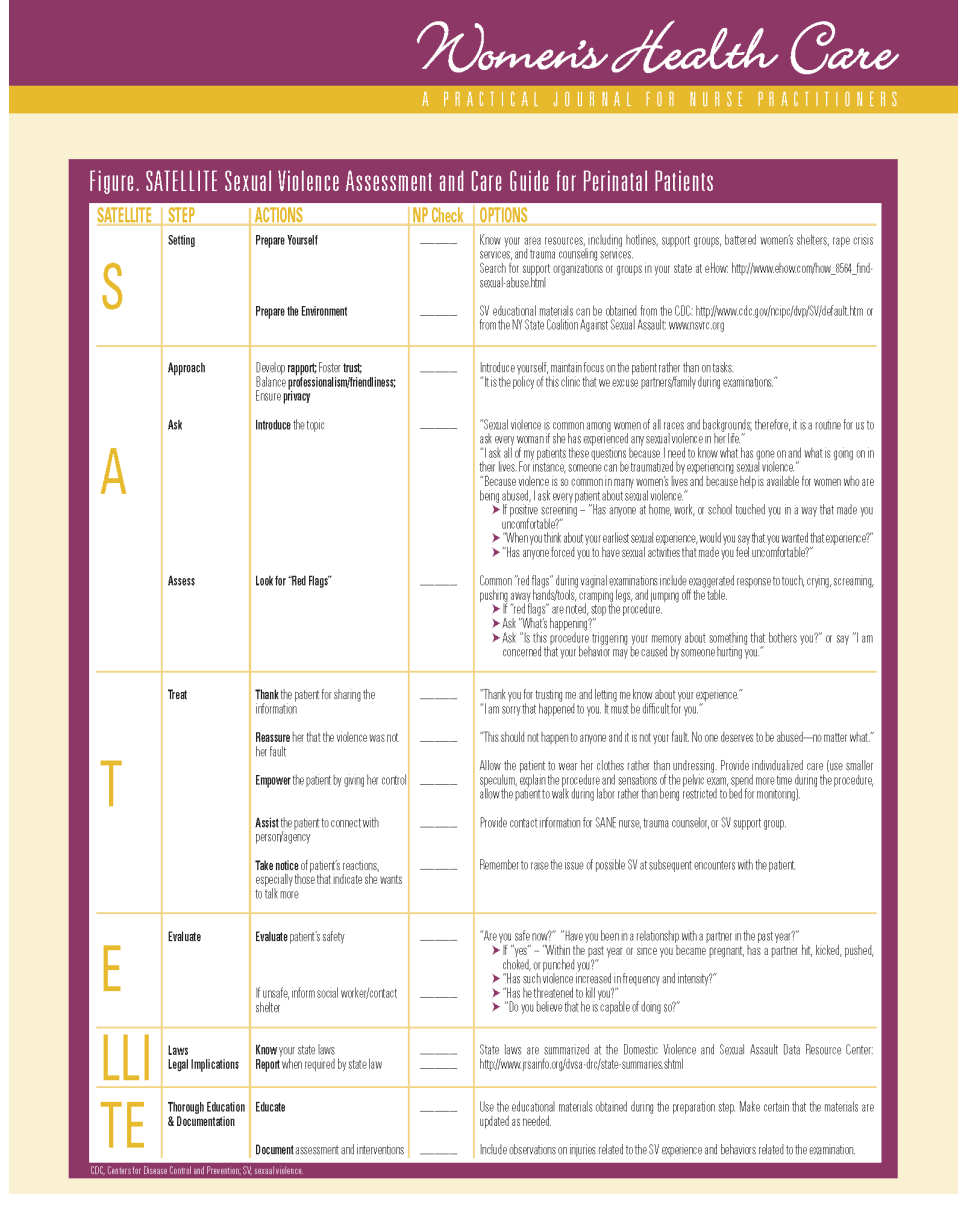
SATELLITE Sexual Violence Assessment and Care Guide: Utilized with permission from Women’s Health Care [26]. Retrieved on 4 May 2024.

The SATELLITE education program comprises PowerPoint slides and presenters. The team followed the CDC’s recommendation [30] to guide the development of the PowerPoint slides, ensuring they encapsulated the learning objectives. This involved incorporating the significance of SV, insights from nurses’ screening experiences for SV based on Ross et al. [24], and the content of the SATELLITE guide [26]. See Figure 1. 

#### 2.3.2. SATELLITE Knowledge/Confidence Instrument

In this study, knowledge and confidence refer to participants’ perceptions of their ability to assess SV and provide appropriate care to SV victims. To evaluate the efficacy of the evidence-based SATELLITE education program, the team examined whether participants’ knowledge and confidence increased after the intervention. The first author developed an 11-item SATELLITE Knowledge/Confidence Instrument (SKI-11) based on the SATELLITE checklist [26]. The instrument underwent review by other team members, with minor wording adjustments made. Additionally, two content experts (a PhD-prepared SV researcher and a Sexual Assault Nurse Examiner) provided feedback, leading to further refinement of the instrument. The finalized SKI-11 was utilized to assess RN participants’ knowledge and confidence before and after the SATELLITE education. Response options for the extent to which participants agree with each statement in the SKI-11 range from “not at all confident” (1) to “very confident” (5), with a total score derived from summing all item scores (potential range 11–55). Higher scores indicate greater knowledge and confidence in SV assessment and appropriate care for SV victims. Demographic questions included participants’ work roles (e.g., midwife, APN, and RN), years of professional experience (open-ended), and prior experience in screening for SV (yes or no).

### 2.4. Data Collection

For optimal delivery of the education, the first and second authors rehearsed their presentations and sought feedback from team members, making necessary adjustments. During the educational session held at the hospital unit, the unit manager introduced the research team. The first author presented the information sheet to 11 RNs present at the start of the session. Each RN was allotted time, if desired, to complete the paper–pencil pre-test SKI-11 with a randomly pre-assigned unique number to facilitate paired t-test data analysis. The PowerPoint presentation of the educational content lasted approximately 40 min, incorporating active learning through questions to prompt participants to discuss and share their experiences, aligned with Knowles’ Andragogy Adult Learning Theory [31,32]. However, due to patient emergencies, three participants had to leave midway through the session. Immediately following the education, the remaining participants (*n* = 8) completed the post-test questionnaire packet and demographic questions. Participants submitted both pre- and post-test surveys in a basket provided at the room exit. Notably, pre-test questionnaires were printed on white paper, while post-test questionnaires were printed on pink paper to distinguish between them, minimizing data collection and data entry errors.

### 2.5. Data Analysis

The completed pre- and post-surveys were matched with the random number assigned to each participant to facilitate data entry for paired t-tests. Data were manually entered using SPSS v. 24 on a password-protected university-owned computer, with accuracy double-checked to ensure precision. No missing data were detected. Descriptive analysis, including frequencies, mean, and standard deviation, was conducted for the three demographic questions. To assess the reliability of the SKI-11, Cronbach’s alpha was computed for internal consistency, and Pearson’s r was calculated for test–retest reliability. Paired t-tests were employed to analyze the differences in scores between pre- and post-SATELLITE education. The significance level was set at 0.05 to determine statistical significance. Cohen-*d*s were generated by the Social Science Statistics Effect Size Calculator for *t*-test [33].

### 2.6. Procedure and Ethical Considerations

The research team collaborated with the Director of the women’s health clinic, located in the southern region of the United States, where monthly educational sessions were conducted for the unit’s nursing staff. The Director facilitated the inclusion of SATELLITE education as part of the unit’s August 2018 monthly meeting, allowing for the provision of continuing education credit. During recruitment, potential risks and benefits of this study along with university Institutional Review Board (IRB) approval, were clearly outlined in the recruitment script. It was emphasized that participation in the educational session was mandatory for unit staff, but completing the pre- and post-test surveys was voluntary and signified consent to participate in the study. After data collection, all data were securely stored in a 2-lock password-protected system to ensure confidentiality. Data analysis was conducted in an aggregated manner to prevent the identification of individual responses.

## 3. Results

Results are presented below based on the objectives of this pilot study.

### 3.1. Feasibility: Can the SATELLITE Education Program Be Implemented as Planned? Is the Instrument Reliable?

The delivery of the SATELLITE education program proceeded successfully as planned, with active participation and constructive discussions from participants. The SKI-11 demonstrated strong internal consistency, with Cronbach’s alpha coefficient of 0.94 for the pre-test and 0.95 for the post-test. Pearson’s r value of 0.53 indicated good test–retest reliability, affirming the reliability of the instrument [34].

### 3.2. Acceptability: Will Key Stakeholders Embrace the Education?

The acceptance of the SATELLITE education program by key stakeholders, including the center director, nurse manager, and RN staff, was evident, as the research team was invited to present the education as a continuing education opportunity at a monthly meeting for all unit staff members. In addition to nursing staff, a physician resident attended the education at the beginning and filled out the pre-test questionnaire but had to leave towards the end due to a patient emergency. All eight participants who attended the session filled out both pre- and post-test questionnaires (along with the demographic form), indicating acceptability of the education program. A request for follow-up trainings and education materials was also made by the center director.

### 3.3. Efficacy: Does the Education Produce the Desired Effects on RNs’ Knowledge and Confidence in SV Assessment and Care Provision for SV Victims?

Paired t-tests demonstrated statistically significant differences in participants’ SKI-11 total scores between pre- and post-tests, with higher scores post-education (t = 3.93, *p* = 0.006). RN participants displayed significantly increased knowledge and greater confidence (*p* < 0.05) with large effect sizes, using post hoc power analysis with a Cohen-*d* result of ≥0.80 [34] in the following areas: how to screen for SV; where to retrieve SV educational materials; where to look for sexual violence support organizations or groups in a geographical area; how to introduce SV topics to patients; how to identify “red flags” for patients who have experienced SV; what to say or do when patients reveal SV experience; knowledge about state laws and regulations related to SV; how to educate patients about SV; and how to document nursing assessment and interventions. Two items of the SKI-11 were not statistically significant (*p* > 0.05): item 5 (how to care for patients who have experienced SV) with a Cohen-*d* of 0.37 (leaning towards a medium effect size of 0.50); and item 8 (how to evaluate the patients’ safety after they reveal their SV experiences) with a Cohen-*d* of 0.76 (leaning towards a large effect size of 0.80) [34]. See Table 1.

Overall, the SATELLITE education program significantly bolstered the knowledge and confidence of RN participants in both screening for and caring for victims of SV. This positive impact was observed across participants, including seasoned professionals with extensive experience in the field, all of whom had prior experience screening patients for SV. The impact of the SATELLITE education program is underscored by the large effect sizes for 9 out of 11 items as measured by the SKI-11. Two items (how to care for SV victims and how to evaluate patients’ safety) were not statistically significant; however, the effect size of these two items leans towards medium to large [34].

### 3.4. Discussion

The SATELLITE guide and education program were developed to equip healthcare providers in overcoming barriers to SV assessment, such as discomfort and reluctance to screen and intervene [26]. Findings from this pilot study demonstrate that the SATELLITE education program is both feasible and acceptable. The SKI-11 pre- and post-test questionnaires, developed through evidence and validated through expert reviews, demonstrated good test–retest reliability and internal consistency. Therefore, the team recommends its continued use as a pre- and post-education assessment tool for evaluating the effects of future SATELLITE education program sessions. 

Results from this study demonstrate notable enhancements in RNs’ knowledge and confidence regarding SV assessment and care, suggesting the efficacy of the SATELLITE education program. Consequently, it is recommended that the SATELLITE education program undergoes evaluation among RNs with a larger sample size across diverse geographic regions and healthcare settings, including rural, urban, for-profit, and nonprofit facilities. 

Moreover, conducting a pilot study to assess the feasibility, accessibility, and efficacy of the SATELLITE education program among other healthcare providers and students, such as physicians, physician assistants, psychologists, and social workers, would be beneficial. Because results from our pilot study were positive among experienced RNs, conducting pilot work to compare results between less experienced and more experienced groups would be beneficial. 

Adjustments to the SATELLITE checklist (Figure 1) and PowerPoint slides are advised for future implementation to ensure alignment with each state-specific SV rates, local resources, and legal considerations. Additionally, the SATELLITE checklist may need to be redesigned in terms of font and design to comply with the Americans with Disabilities Act. This consideration arises from the possibility that the font in its original format may be too small for accessibility. Furthermore, adapted versions of the SATELLITE guide for use in intimate partner violence screening should be considered. Expansion of the SATELLITE guide to encompass a broader spectrum of genders is also recommended.

The acceptability and positive impact on confidence and knowledge resulting from the in-person session suggest potential merit in this approach. A valuable next step would involve comparing the measures obtained in-person with those from other options (such as online self-paced learning or simulation. Utilizing the CDC’s Training of Trainers model (ToT) for future studies could streamline effective presentation of educational content [35]. Further research should inquire about ways to improve the education program and evaluate participants’ application of learned skills upon returning to work (learning transfer) through open-ended questions or in-depth interviews [36].

Although data were collected in 2018, this pilot study fills a gap in research on the feasibility, acceptability, and efficacy of the SATELLITE education program. These study results are worthwhile as foundational information for future larger studies. Sharing data from expanded implementations of the program can enhance its effectiveness and support evidence-based practice among nurses and other healthcare providers in SV assessment and care. Equipping nurses and other healthcare professionals, including students, with the knowledge and confidence gained from SATELLITE education is crucial for providing equitable and effective care to survivors of SV. 

A couple limitations should be considered in this study. First, literature shows that while screening seems to increase disclosures, it does not necessarily lead to help-seeking or improved outcomes for survivors of violence [19,22,23,37]. Therefore, healthcare providers need to consider survivors’ needs and unique context to ensure that they are not in danger, while reducing suffering and upholding their human rights [15]. Second, the SKI-11 is a self-assessment tool that measures the program’s short-term effectiveness. The translation of learning to practice should be incorporated in future research.

In conclusion, this pilot study effectively addresses a significant research gap and advances evidence-based practice in SV assessment and care. The study findings confirm the efficacy of the SATELLITE education program in enhancing RN knowledge and confidence, as well as the value of the SKI-11 pre- and post-test questionnaires as an assessment tool. Future testing of the SATELLITE education program should encompass a broader scope, with refined program materials delivered to both undergraduate and graduate nursing students as well as across diverse healthcare settings and disciplines. The SATELLITE education program holds immense potential to positively impact the lives of SV survivors, and its ongoing implementation and refinement are essential for fostering compassionate care within healthcare settings and beyond.

## Figures and Tables

**Table 1:** Results of paired *t*-test of mean scores before and after SATELLITE education along with Cohen-*d* effect sizes.

Item	Before M (SD)	After	*t*	*p*	*Cohen-d*
1. I know how to screen for my patients’ sexual violence experiences.	3.38 (1.06)	4.50 (0.53)	3.22	0.015 **	1.34 (large)
2. I know where to look for sexual violence support organizations or groups in my geographical area.	3.25 (1.03)	4.75 (0.46)	3.97	0.005 **	1.56 (large)
3. I know where to retrieve sexual violence educational materials.	3.25 (0.88)	4.88 (0.35)	5.02	0.002 **	2.41 (large)
4. I can introduce sexual violence topics to my patients.	3.63 (1.06)	4.38 (0.51)	2.39	0.048 **	0.90 (large)
5. I can care for patients who have experienced sexual violence.	3.75 (1.28)	4.13 (0.64)	1.16	0.285	0.37 (~towards medium)
6. I know what “red flags” are for patients who have experienced sexual violence.	3.25 (1.03)	4.38 (0.51)	3.81	0.007 **	1.37 (large)
7. I know what to say or do when my patients reveal their sexual violence experiences.	3.31 (1.16)	4.25 (0.46)	2.62	0.034 *	1.07 (large)
8. I know how to evaluate my patients’ safety after they reveal their sexual violence experiences.	3.50 (1.19)	4.25 (0.70)	1.82	0.111	0.76 (~large)
9. I know my state laws and regulations related to sexual violence.	2.88 (0.64)	4.00 (0.75)	3.21	0.015 *	1.61 (large)
10. I know how to educate my patients about sexual violence.	3.00 (1.06)	4.25 (0.462)	3.04	0.019 *	1.52 (large)
11. I know how to document my patients’ sexual violence experiences.	3.13 (0.99)	4.25 (0.70)	4.97	0.002 **	1.31 (large).

Note: Cohen-*d*s were generated by Social Science Statistics Effect Size Calculator for *t*-test [33]. * *p* < 0.05; ** *p* < 0.01.

## Data Availability

Data is unavailable due to privacy or ethical restrictions.

## Public Involvement Statement

No public involvement in any aspect of this research.

## Guidelines and Standards Statement

This manuscript was drafted against the CONSORT2010 Statement (https://www.equator-network.org/reporting-guidelines/consort/ (accessed on 25 February 2024)) for original research.

## References

[1] Rape, Abuse, & Incest National Network (2024). Types of Sexual Violence.

[2] Centers for Disease Control and Prevention (2022). Fast Facts: Preventing Sexual Violence.

[3] International Justice Mission (2024). The Problem: Sexual Violence.

[4] World Health Organization (2021). Devastatingly Pervasive: 1 in 3 Women Globally Experience Violence.

[5] Rape, Abuse, & Incest National Network (2024). Victims of Sexual Violence: Statistics.

[6] Ross R., Stidham A.W., Saenyakul P., Creswell J.W. (2015). Intimate partner violence, emotional support, and health outcomes among Thai women: A mixed methods study. J. R. Thai Army Nurses.

[7] Leemis R.W., Friar N., Khatiwada S., Chen M.S., Kresnow M., Smith S.G., Caslin S., Basile K.C. (2022). The National Intimate Partner and Sexual Violence Survey: 2016/2017 Report on Intimate Partner Violence.

[8] Farahi N., McEachern M. (2021). Sexual assault of women. Am. Fam. Physician.

[9] Peterson C., DeGue S., Florence C., Lokey C. (2017). Lifetime economic burden of rape among U.S. adults. Am. J. Prev. Med..

[10] Hailes H.P., Yu R., Danese A., Fazel S. (2019). Long-term outcomes of childhood sexual abuse: An umbrella review. Lancet Psychiatry.

[11] Holliday R., Nichter B., Holder N., Hill M.L., Monteith L.L., Norman S.B., Pietrzak R.H. (2023). Childhood sexual abuse and military sexual trauma interact to increase suicide risk: Results from a nationally representative veteran sample. J. Interpers. Violence.

[12] Strizzi J.M., Mortensen E.L., Hegelund E.R., Wimmelmann C.L., Folker A.P., Flensborg-Madsen T. (2022). Experience of sexual violence and satisfaction with life: A 20-year prospective cohort study. J. Sex. Aggress..

[13] Letourneau E.J., Brown D.S., Fang X., Hassan A., Mercy J.A. (2018). The economic burden of child sexual abuse in the United States. Child Abus. Negl..

[14] The National Academies of Sciences, Engineering, and Medicine (2021). The Future of Nursing 2020–2030: Charting a Path to Achieve Health Equity.

[15] American Nurses Association (2021). Nursing: Scope and Standards of Practice.

[16] (2019). ACOG Committee Opinion No. 777: Sexual Assault. Obstet. Gynecol..

[17] Baird K., Creedy D.K., Saito A.S., Eustace J. (2018). Longitudinal evaluation of a training program to promote routine antenatal enquiry for domestic violence by midwives. Women Birth.

[18] Poreddi V., Gandhi S., Reddy S.N., Palaniappan M., BadaMath M. (2020). Effectiveness of nurses training in routine screening of violence against women with mental illness: A randomized controlled trail. Arch. Psychiatr. Nurs..

[19] Miller C.J., Adjognon O.L., Brady J.E., Dichter M.E., Iverson K.M. (2021). Screening for intimate partner violence in healthcare settings: An implementation-oriented systematic review. Implement. Res. Pract..

[20] Basile K.C., Hertz M.F., Back S.E. (2016). Intimate Partner Violence and Sexual Violence Victimization Assessment Instruments for Use in Healthcare Settings: Version 1.

[21] Tavrow P., Bloom B.E., Withers M.H. (2017). Intimate partner violence screening practices in California after passage of the Affordable Care Act. Violence Against Women.

[22] Feder G., Ramsay J., Dunne D., Rose M., Arsene C., Norman R., Kuntze S., Spencer A., Bacchus L., Hague G. (2009). How far does screening women for domestic (partner) violence in different health-care settings meet criteria for a screening programme? Systematic reviews of nine UK National Screening Committee criteria. Health Technol. Assess..

[23] O’doherty L.J., Taft A., Hegarty K., Ramsay J., Davidson L.L., Feder G. (2014). Screening women for intimate partner violence in health care settings: Abridged Cochrane systematic review and meta-analysis. BMJ.

[24] Ross R., Draucker C.B., Martsolf D., Adamle K., Chiang-Hanisko L., Lewandowski W. (2010). The bridge: Providing nursing care for survivors of sexual violence. J. Am. Acad. Nurse Pract..

[25] American Academy of Physician Assistants (2020). PA Training and Skills to Work with Survivors of Sexual Violence: Trends and Implications for PAs. https://www.aapa.org/wp-content/uploads/2020/04/PA-Training-and-Skills-to-Work-With-Survivors-of-Sexual-Violence.pdf.

[26] Ross R., Roller C., Rusk T., Martsolf D., Draucker C. (2009). The SATELLITE sexual violence assessment and care guide for perinatal patients. Women’s Health Care.

[27] Cash J.C., Glass C.A. (2023). Adult-Gerontology Practice Guidelines.

[28] Cash J.C., Glass C.A. (2017). Family Practice Guidelines.

[29] Ward S.L., Hisley S.M., Kennedy A.M. (2015). Maternal-Child Nursing Care: Optimizing Outcomes for Mothers, Children, and Families.

[30] Centers for Disease Control and Prevention (2019). Understanding the Training of Trainers Model.

[31] Knowles M. (1990). The Adult Learners: A Neglected Species.

[32] New England Institute of Technology (2021). What Is Adult Learning Theory?.

[33] Strangroom J. (2021). Social Science Statistics Effect Size Calculator for T-test. https://www.socscistatistics.com/effectsize/default3.aspx.

[34] Hair J.F., Black W.C., Babin B.J., Anderson R.E. (2010). Multivariate Data Analysis.

[35] Centers for Disease Control and Prevention (2021). Training Effectiveness.

[36] Centers for Disease Control and Prevention (2020). Violence Prevention: Preventing Sexual Violence.

[37] Ramsey J., Rivas C., Feder G. (2005). Interventions to Reduce Violence and Promote the Physical and Psychosocial Well-Being of Women Who Experience Partner Abuse: A Systematic Review of Controlled Evaluations.

